# Modulation of calcium signaling pathway by hepatitis C virus core protein stimulates NLRP3 inflammasome activation

**DOI:** 10.1371/journal.ppat.1007593

**Published:** 2019-02-27

**Authors:** Amina A. Negash, Rebecca M. Olson, Stephen Griffin, Michael Gale

**Affiliations:** 1 Center for Innate Immunity and Immune Disease, Department of Immunology, University of Washington School of Medicine, Seattle, Washington, United States of America; 2 School of Medicine, Faculty of Medicine and Health, University of Leeds, St James’ University Hospital, Leeds, United Kingdom; Nationwide Children's Hospital, UNITED STATES

## Abstract

Hepatitis C virus (HCV) infection remains a major cause of hepatic inflammation and liver disease. HCV triggers NLRP3 inflammasome activation and interleukin-1β (IL-1β) production from hepatic macrophages, or Kupffer cells, to drive the hepatic inflammatory response. Here we examined HCV activation of the NLRP3 inflammasome signaling cascade in primary human monocyte derived macrophages and THP-1 cell models of hepatic macrophages to define the HCV-specific agonist and cellular processes of inflammasome activation. We identified the HCV core protein as a virion-specific factor of inflammasome activation. The core protein was both necessary and sufficient for IL-1β production from macrophages exposed to HCV or soluble core protein alone. NLRP3 inflammasome activation by the HCV core protein required calcium mobilization linked with phospholipase-C activation. Our findings reveal a molecular basis of hepatic inflammasome activation and IL-1β release triggered by HCV core protein.

## Author summary

This study deciphers the molecular mechanism of Hepatitis C virus (HCV)-induced hepatic inflammation. HCV triggers NLRP3 inflammasome activation and IL-1β release from hepatic macrophages, thus driving liver inflammation. Using biochemical, virological, and genetic approaches we identified the HCV core protein as the specific viral stimulus that triggers intracellular calcium signaling linked with phospholipase-C activation to drive NLRP3 inflammasome activation and IL-1β release in macrophages.

## Introduction

HCV continues as a global health problem causing chronic and progressive liver disease [1–5]. HCV is a major risk factor for hepatocellular carcinoma, and infection is a consistent cause of liver transplants. HCV is a small, enveloped, single-stranded RNA virus that belongs to the *Flaviviridae* family [6]. It is transmitted through parenteral routes and replicates primarily in the liver. Most often, exposure to HCV leads to chronic infection, which is characterized by persistent hepatic inflammation. The hallmark of chronic HCV infection is dysregulated and persistent inflammatory responses that are thought to serve as a platform for ongoing liver damage and the onset of cirrhosis and hepatocellular carcinoma [7]. While currently no vaccine for HCV is available for clinical use, the advent of direct acting antivirals (DAAs) has revolutionized patient care and these drugs are proven to be effective treatment options for HCV infected individuals beyond interferon (IFN)-based therapy [8, 9]. DAAs are oral regimens, well-tolerated and most patients achieve 80–90% sustained virologic responses (SVRs, defined as the absence of HCV RNA detection after cessation of treatment with DAAs). However, with DAAs there is a concern of the emergence of drug resistant HCV variants, the unknown effects of drug-to-drug interactions, and the expensive nature of these drugs [10, 11]. Most importantly, further prospective studies are needed to assess the effects of treatment with DAAs on preventing liver fibrosis and mitigating HCV-induced severe liver disease such as HCC [12, 13]. Therefore, understanding the complete molecular mechanism of HCV-induced hepatic inflammation is essential to design the best therapeutic regimen to treat hepatic inflammation and to reduce liver damage resulting from chronic HCV infection.

HCV replicates in hepatocytes, the chief parenchymal cell of the liver. During infection HCV also interacts with hepatic macrophages such as the liver-resident Kupffer cells (KCs), which make up 15–20% of the hepatic non-parenchymal cells [14]. KCs are highly phagocytic and play an important dual role within the hepatic microenvironment. They maintain hepatic homeostasis during immune responses to liver injury and also function as central mediators of hepatic inflammation induced in response to microbial-derived products [14–16]. The inflammatory cascade within the liver is initiated and propagated by KCs upon recognition of danger-associated molecular patterns (DAMPs) such as HMGB1 and pathogen-associated molecular patterns (PAMPs) such as viral RNA and/or viral proteins [17, 18]. Activated KCs produce and secrete a diverse array of chemokines and cytokines leading to leukocyte recruitment to the liver.

One of the key intrahepatic inflammatory soluble factors produced by KCs in response to DAMP or PAMP interaction is interleukin-1β (IL-1β) [19]. IL-1β is a potent proinflammatory cytokine that induces the production of chemokines and cytokines such as CXCL4, TNF and IL-6. IL-1β production by hepatic macrophages leads to the recruitment and activation of myeloid cells and lymphocytes in the liver [20–23]. Importantly, IL-1β plays a pivotal role to modulate the immune response during both acute and chronic virus infection [24–27]. While IL-1α, a closely related cytokine to IL-1β, signals through the same receptor [28], it is widely expressed by many cells in direct response to stimuli that activate NF-κB [29]. In contrast, IL-1β production is tightly regulated through a two-step process of IL-1β expression and inflammasome activation. The production of a bioactive IL-1β cytokine requires the assembly of a cytoplasmic multiprotein complex called the inflammasome [30, 31]. This multimeric complex is typically composed of at least three proteins: a nucleotide-binding oligomerization domain-like receptor (NOD-like receptor) such as NLRP3, the adaptor protein ASC and the effector protease caspase-1. IL-1β production requires a priming signal or (signal-one) initiated by DAMP and/or PAMP recognition and signaling by the responding cell to drive the production of inactive, proIL-1β protein. An inflammasome-activating signal or (signal-two) is then required to recruit and assemble the inflammasome components with ASC and procaspase-1 leading to caspase-1 activation, cleavage of pro-IL-1β to mature form and the release of active IL-1β protein. The NLRP3 inflammasome is one of the well-studied inflammasomes that is activated by diverse stimuli including RNA viruses [32, 33]. Assembly of the NLRP3 inflammasome is triggered and governed by integrating diverse activating signals such as calcium mobilization and influx, potassium efflux, reactive oxygen species and/or by interaction with cellular factors such as NEK7 [34–36].

Elevated serum levels of IL-1β and IL-18 are prevalent in patients infected with HCV [27, 37]. Furthermore, IL-1β is expressed exclusively within the liver of patients with cirrhosis, but not within a normal liver or an HCV-infected liver exhibiting no fibrosis/disease [27, 37]. These findings provide strong evidence linking IL-1β with HCV-induced hepatic inflammation and disease. HCV interaction with macrophages triggers IL-1β production and release through NLRP3 inflammasome activation [19, 27, 33]. In these studies, HCV was shown to stimulate both immature and mature IL-1β production, indicating that one or more of the HCV virion components provides the necessary signals to stimulate NLRP3 inflammasome from within macrophages. HCV induces signal-one of NLRP3 inflammasome activation through viral RNA triggering and signaling through Toll-like receptor (TLR)7 [27, 33], but how it imparts signal-two to drive inflammasome assembly and activation are not known. In this current study, we sought to identify the HCV-specific component(s) that stimulate NLRP3 inflammasome activation and the production of IL-1β to define the molecular mechanism of hepatic inflammation directed by IL-1β and induced by HCV. Our study identifies virion core protein as the major specific NLRP3 inflammasome agonist that drives inflammasome assembly leading to the production/release of bioactive IL-1β from macrophages. We show that the viral core protein directs intracellular calcium mobilization to impart NLRP3 inflammasome assembly through activation and signaling of phospholipase-C. Our study reveals a pivotal role of virion-associated and soluble/circulating core protein in HCV-induced hepatic inflammation, underscoring the contribution of the viral core protein in HCV pathogenesis and liver disease [38–43].

## Results

**Fig 1:**
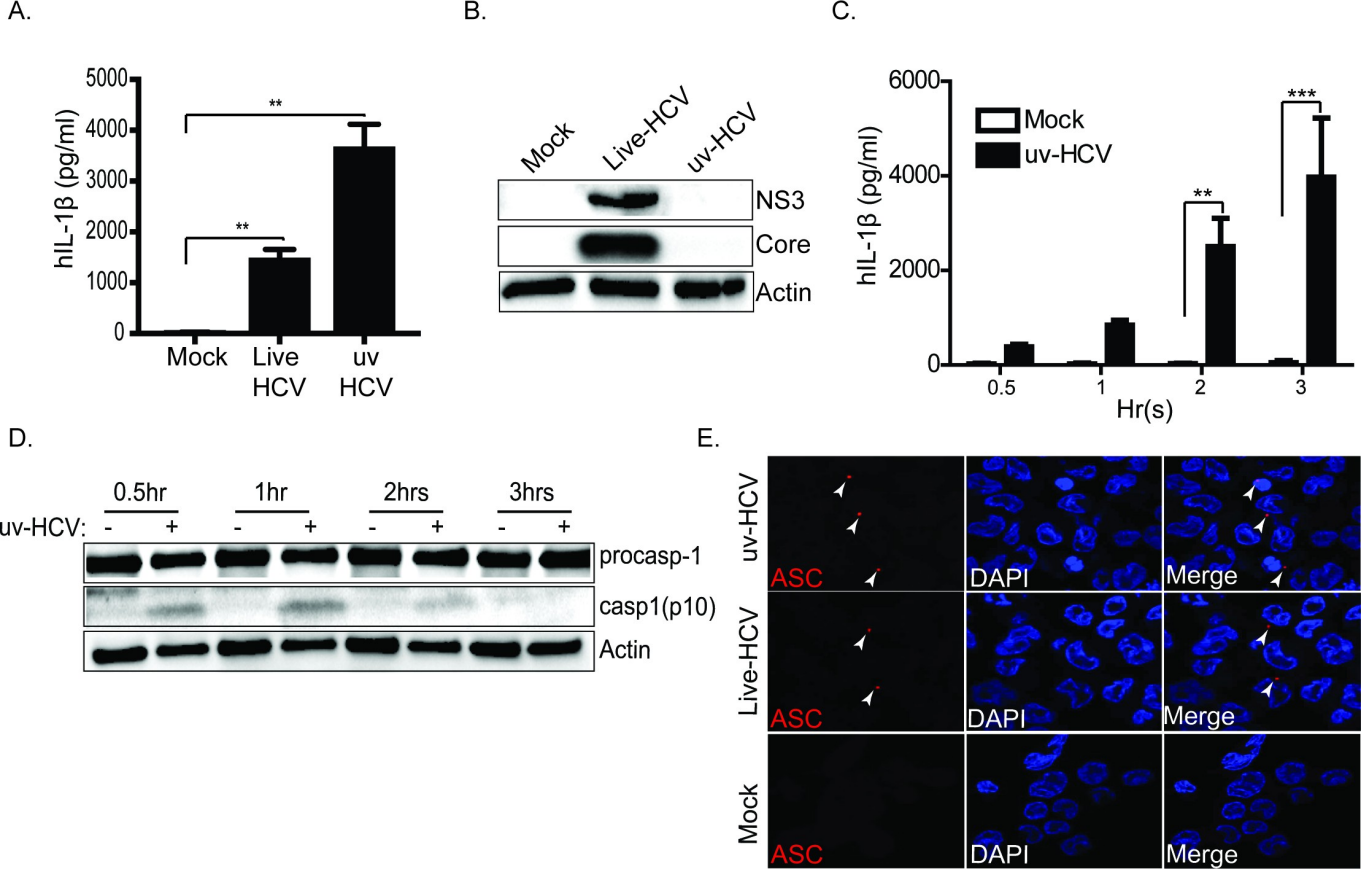
Component(s) within the incoming HCV virion stimulate NLRP3 inflammasome activation and IL-1β production. (A) IL-1β ELISA in THP-1 cells. THP-1 cells were differentiated with PMA overnight then rested for another 24hrs. The cells were exposed to infectious supernatant containing live-HCV or HCV inactivated by ultraviolet irradiation (uv-HCV) then the response was monitored. (B) immunoblot showing the replication capacity of uv-HCV as compared to live-HCV. (C) Differentiated THP-1 cells were exposed to infectious supernatant containing HCV inactivated by ultraviolet irradiation (uv-HCV) then the response was monitored over the indicated time course. (D) immunoblot showing the processing of caspase-1 post exposure to uv-HCV. Actin serves as a loading control. (E) ASC-specks in uv-inactivated HCV or live-HCV treated THP-1 cells. Differentiated THP-1 cells were stimulated with uv-HCV/live-HCV for 1hr then fixed with paraformaldehyde. Fixed cells were stained with anti-ASC antibody and DAPI. Red stains ASC-specks and DAPI stains the nuclei. ASC specks are indicated by the arrows. Experiments were performed with at least two technical replicates and are representative of three independent experiments. Data are presented as means +/- SD. *P<0.05, **P<0.01 and ***P<0.001.

HCV is a potent inducer of IL-1β production in macrophages and our studies have shown that HCV itself contains all the factors needed to trigger both signal-one and signal-two of NLRP3 inflammasome activation [27, 33]. To determine which of the HCV virion component(s) is (are) essential for stimulating NLRP3 inflammasome activation, we prepared HCV and subjected it to ultraviolet (uv) light, resulting in inactivated HCV (uv-HCV) (Fig 1B). Uv-HCV retains the ability to bind and enter cells but is unable to replicate [27]. THP-1 macrophages produce active IL-1β after exposure to uv-HCV (Fig 1A and 1C) coincident with the rapid processing and activation of caspase-1 (Fig 1D) and the formation of ASC- specks indicative of inflammasome activation (Fig 1E). These results collectively reveal that the components of the inactivated HCV virion, containing the viral RNA and structural proteins, but not the viral non-structural protein(s) [44], serve as the NLRP3 inflammasome agonist to drive caspase-1 processing, ASC-speck formation and IL-1β release from macrophages exposed to HCV. Thus, while viral replication is not required for inflammasome activation, components of the incoming virion must deliver the necessary signals, both to prime (signal-one) and activate the NLRP3 inflammasome (signal-two).

**Fig 2:**
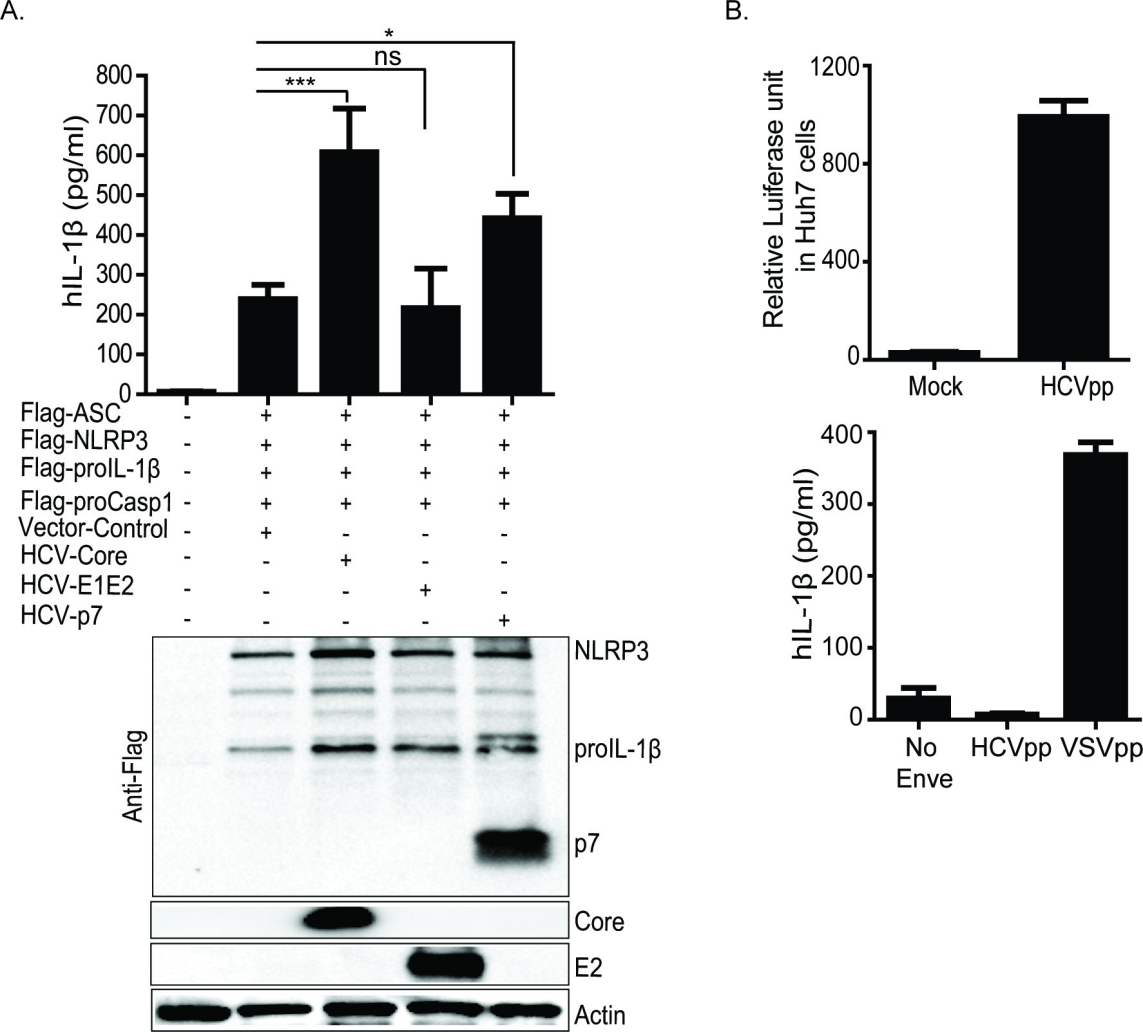
HCV capsid protein and ion-channel p7, but not envelope proteins (E1 and E2) activate NLRP3 inflammasome signaling. (A) IL-1β ELISA (top panel) in U2OS cells reconstituted with NLRP3 inflammasome components. Human proIL-1β, procasp1, ASC and NLRP3 were co-transfected with constructs expressing vector only or HCV core or HCV-p7 or HCV-E1E2. Lower panel of (A) is a western blot depicting the expression of each transfected constructs including the loading control, actin. (B) Relative luciferase activity measured in hepatoma Huh7 cells post infection with HCVpp (upper panel). Lower panel (B), IL-1β ELISA in THP-1 cells stimulated with (HCVpp) or VSVpp. Experiments were performed with replicates and are representative of at least two independent experiments. Data are presented as means +/- SD. *P<0.05, ***P<0.001 and ns = non-significant.

Ten proteins are encoded by the HCV virion wherein three of these proteins, Core, E1 and E2, are the viral structural proteins [44]. HCV core protein encapsulates the viral RNA and it is essential for viral assembly whereas the viral glycoproteins, E1 and E2, are the envelope proteins involved in entry [45]. E1 and E2 co-exist as a heterodimer via non-covalent interaction in which E2 is essential for proper function of E1. In addition to the structural proteins, HCV encodes a small ion-channel protein p7 and six non-structural proteins (NS2, NS3, NS4A, NS4B, NS5A and NS5B). Although p7 protein is essential for both virion assembly and egress in hepatocytes, it is unclear to this date if p7 is a true component of the mature HCV virion [46]. We evaluated the role of each structural protein and p7 in NLRP3 inflammasome activation using the NLRP3 inflammasome reconstitution [47] system in U2OS (human osteosarcoma) cells. In this cell system, the NLRP3 inflammasome components of NLRP3, ASC, procaspase-1, and proIL-1β are ectopically co-expressed, thus bypassing the requirement for signal-one to drive the expression of inflammasome components but are able to respond to a signal-two stimulus for inflammasome assembly and activity. We first co-expressed each HCV structural protein or p7 with the NLRP3 inflammasome components in U2OS cells and examined the ability of each viral protein to drive inflammasome activation marked by induction of mature IL-1β release as compared to cells co-expressing vector control. We found that the HCV core protein, and to a lesser extent the viral p7 protein, but not the envelope glycoproteins E1-E2, were able to stimulate NLRP3 inflammasome activation (Fig 2A). To further assess a possible role for E1-E2 in inflammasome activation, we evaluated the ability of HCV pseudoparticles containing E1-E2 (HCVpp) [48] to activate the NLRP3 inflammasome in THP-1 macrophages. HCVpp are viable and able to infect Huh7 [49] hepatoma cells as measured by luciferase activity encoded by the HCVpp (Fig 2B upper panel). THP-1 cells treated with HCVpp did not release IL-1β, but cells treated with vesicular stomatitis virus G protein pseudoparticles (VSVpp; control) produced and released IL-1β, consistent with VSV-mediated inflammasome activation [26] (Fig 2B lower panel).

The HCV ion channel p7, is a small transmembrane protein [46]. Although it remains to be determined if p7 is truly a component of the HCV virion, p7 has been shown to stimulate a level of NLRP3 inflammasome activity, consistent with our observations and others (see Fig 2A and [50]). The differential magnitude of IL-1β production induced by the p7 and core protein suggests that p7 might not be the primary or sole activator of the NLRP3 inflammasome by the HCV virion if indeed it is a virion component. To further examine the role of p7 and its activity as an ion channel in inflammasome activation, we treated THP1 cells with uv-HCV in the presence of a small molecule inhibitor of the p7 ion channel, JK3/32 [51]. As a parallel control, we also treated cells with an inactive compound analog, R-21, that does not inhibit p7. Treatment with either compound had no effect on IL-1β production and release from cells in response to uv-HCV (S1 Fig). We confirmed that the THP1 cells responded to LPS/Nigericin (Ng) treatment to stimulate IL-1β production and release in the presence of each compound (S1A Fig), though p7 inhibitor treatment suppressed HCV infection in Huh7 cells (S1B Fig). Thus, while we confirm that p7 can independently direct a level of inflammasome activation in the reconstituted system, in the context of the HCV virion p7 activity does not play a primary role in inflammasome activation in macrophages. Together, our results reveal the viral core protein as the major component of the HCV virion that induces NLRP3 inflammasome activation leading to the processing and release of IL-1β.

**Fig 3:**
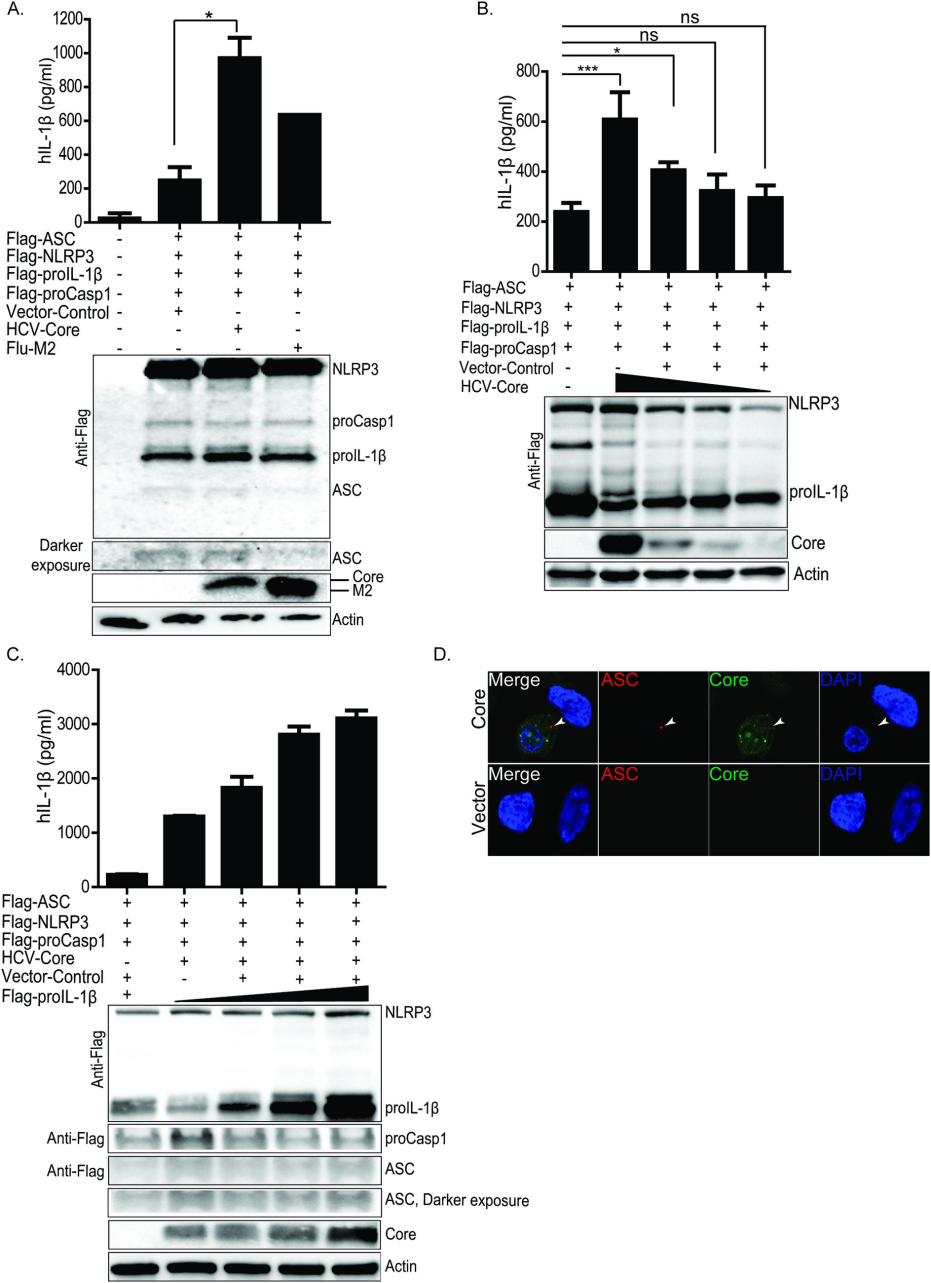
HCV core protein efficiently mediates NLRP3 activation and IL-1β release in the reconstituted system. (A) ELISA of IL-1β in U2OS cells reconstituted with NLRP3 inflammasome components. Top panel of (A) shows ELISA in vector or core or Flu-M2 transfected cells. Lower panel of (A) is IP-western blot depicting the expression of each NLRP3 inflammasome components (ASC, procasp-1, proIL-1β and NLRP3) and the co-transfected vector or core or Flu-M2. (B) ELISA (upper panel) in U2OS cells reconstituted with NLRP3 inflammasome components and transfected with a decreasing doses of HCV core protein expressing construct (western blot, lower panel). (C) ELISA revealing increase in IL-1β release with increasing input of proIL-1β expressing construct (upper panel) and input protein (lower panel) in the reconstituted cells. (D) ASC-specks in vector or core expressing reconstituted cells. Green stains HCV core protein, red stains ASC-specks and blue stains the nuclei. Experiments were performed with more than two replicates and are representative of three independent experiments. Data are represented as means +/- SD. For (A) *P = 0.0113. For (B) *P<0.05 and ***P<0.001.

We examined the ability of HCV core protein to stimulate IL-1β production and ASC-speck formation in the reconstituted system. We found the HCV core protein, as similar to the influenza A virus M2 protein (Flu-M2)- a known NLRP3 inflammasome signal-two viral protein stimulus [25], triggers inflammasome activation in reconstituted U2OS cells. HCV core induced the production of IL-1β to similar or greater extent as Flu-M2 (Fig 3A). HCV core induced IL-1β release in a dose-dependent manner (Fig 3B) and supported high level of IL-1β release when cells received increasing input of proIL-1β cDNA construct (Fig 3C). The presence of HCV core protein caused the appearance of ASC-specs in the reconstituted cells, but not in neighboring nonreconstituted cells (Fig 3D). These results demonstrate that HCV core protein stimulates canonical NLRP3 inflammasome activation to mediate ASC-spec formation and processing and release of IL-1β.

**Fig 4:**
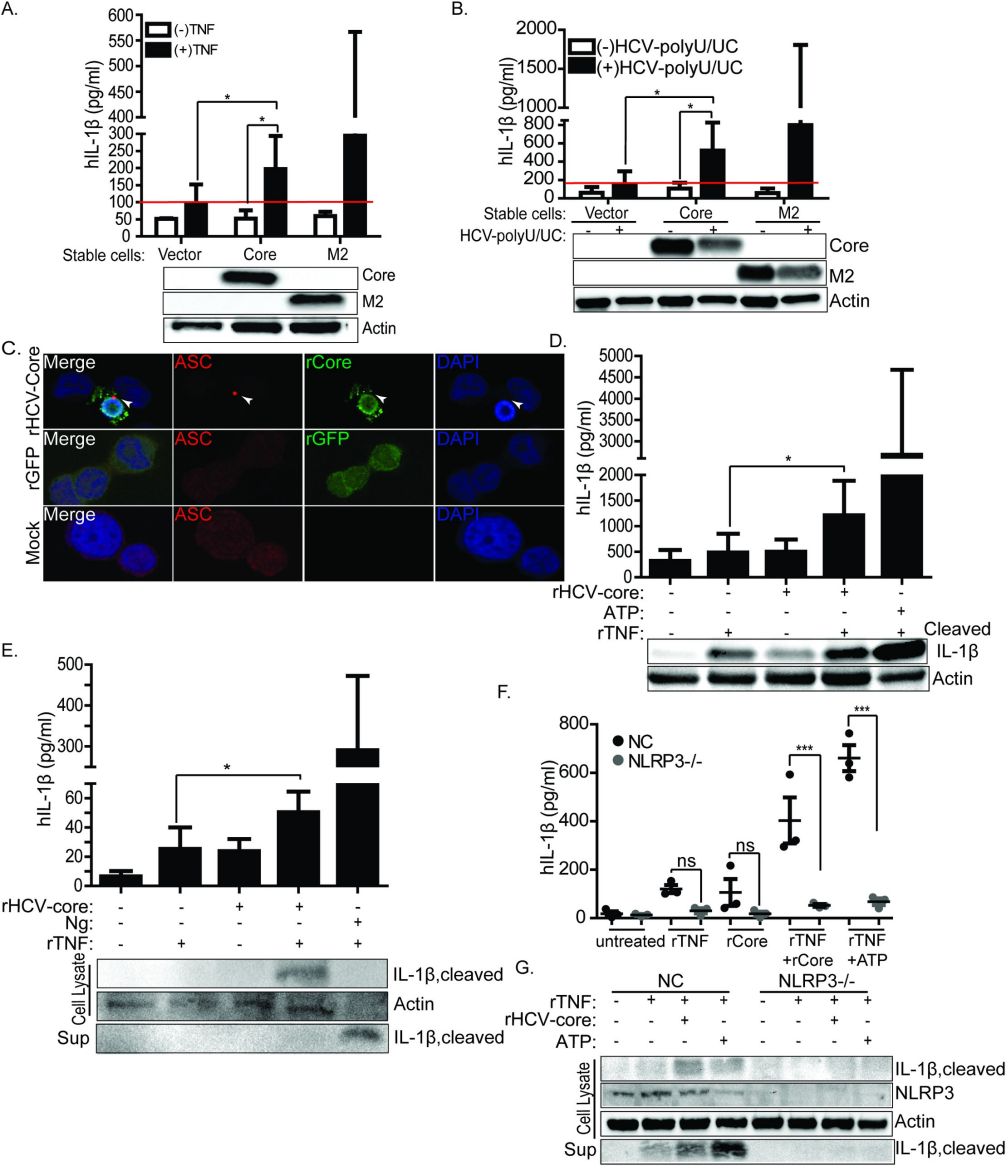
HCV core protein induces IL-1β release in macrophages. (A) IL-1β ELISA in THP-1 cells stably expressing vector only or HCV-core or Flu-M2 post differentiation with PMA. To prime and stimulate proIL-1β expression, cells were stimulated with either recombinant TNF (rTNF) or in (B) transfected with HCV polyU/UC RNA PAMP. Lower panel of (A) and (B) show the expression of core or Flu-M2 in the stable cell lines. (C) ASC-specks in THP-1 cells treated with recombinant core (rHCV-core) or recombinant GFP (rGFP). Red stains ASC-specks, green stains core or rGFP, and blue stains nuclei. ASC-specks are denoted by the white arrows. (D) ELISA showing the release of IL-1β in TNF-primed THP-1 treated with recombinant core (rHCV-core) or ATP. Top panel of (D) shows the release of IL-1β in supernatant and lower panel of (D) shows by western blot cleaved IL-1β protein in each treatment condition. (E) Primary human monocyte-derived macrophages were primed with rTNF for signal-one then treated with rHCV-Core or Ng. Upper panel of (E) is IL-1β ELISA and the lower panel shows production of cleaved of IL-1β both in supernatant and lysate. (F) ELISA of IL-1β in THP-1 non-targeting control cells or THP1 cells lacking NLRP3. These cells were primed with rTNF then stimulated with rHCV-core. (G) western blot depicting the production or lack of cleaved IL-1β in NLRP3 knockout or non-targeting control (NC) cells. Experiments were performed with two replicates and are representative of three independent experiments. Data are represented as means and +/- SD. For (A) *P = 0.0228 and *P = 0.0398, for (B) *P = 0.0317 and *P = 0.0495, for (D) *P = 0.0114, for (E) *P = 0.0178, for (F) ***p<0.001 and ns = non-significant.

To determine if the HCV core protein is a signal-two stimulus for NLRP3 inflammasome activation as occurs in macrophages exposed to HCV [19, 33], we assessed the ability of core protein to induce inflammasome activation in THP-1 macrophages. We generated THP-1 cells stably expressing either vector only, HCV core or Flu-M2 (control). When primed with TNF-α as a signal-one agonist (S2 Fig), each THP-1 macrophage cell line released IL-1β (Fig 4A). TNF-α priming triggered a small and non-significant level of IL-1β in vector-expressing cells as compared to unprimed cells. On the other hand, in core-expressing cells we observed a significant enhancement of IL-1β release as compared to unprimed cells or the control primed-vector-expressing cells. Treatment of cells with poly-U/UC RNA from the HCV genome, a known PAMP and activator of innate immune signaling through retinoic acid inducible gene-I (RIG-I) [52], also primed the cells to facilitate a significant level of IL-1β release from cells expressing core but not vector alone (Fig 4B). Virion-free soluble HCV core protein is readily detected at high concentration in the plasma of HCV-infected individuals [53, 54]. We therefore tested the ability of purified, recombinant HCV core protein (rHCV-core) to stimulate NLRP3 activation in THP-1 cells. We assessed the formation of ASC-specks in cells treated with rHCV-core or purified recombinant green fluorescence protein (rGFP, control). As phagocytic cells, THP-1 macrophages readily internalize cell-free macromolecules in a fashion similar to KCs [27, 55], and immunofluorescence staining of recombinant core or fluorescence analysis of rGFP in cells demonstrated the uptake of each protein by treated THP-1 macrophages. We found that rHCV-core but not rGFP triggered ASC-speck formation after cell uptake (Fig 4C). Treatment of THP-1 cells with rHCV-core following signal-one priming with TNF-α stimulated robust IL-1β production and processing as did ATP, a known signal-two agonist of the NLRP3 inflammasome (Fig 4D). We next confirmed these findings in primary human monocyte-derived macrophages. As in THP-1 cells, TNF-α-primed macrophages produce IL-1β when stimulated with rHCV-core (Fig 4E) as the control cells stimulated with nigericin (Ng), a known activator of the NLRP3 inflammasome [56]. These observations demonstrate that HCV core protein uptake by macrophages undergoing signal-one priming stimulates inflammasome activation to trigger IL-1β production. Further, we confirmed that rHCV-core-induced IL-1β release requires NLRP3 inflammasome-dependent signaling (Fig 4F and 4G). Taken together, these observations imply that both cell-free HCV core protein and virion-associated HCV core protein serve as viral protein agonists of NLRP3 inflammasome activation in macrophages.

**Fig 5:**
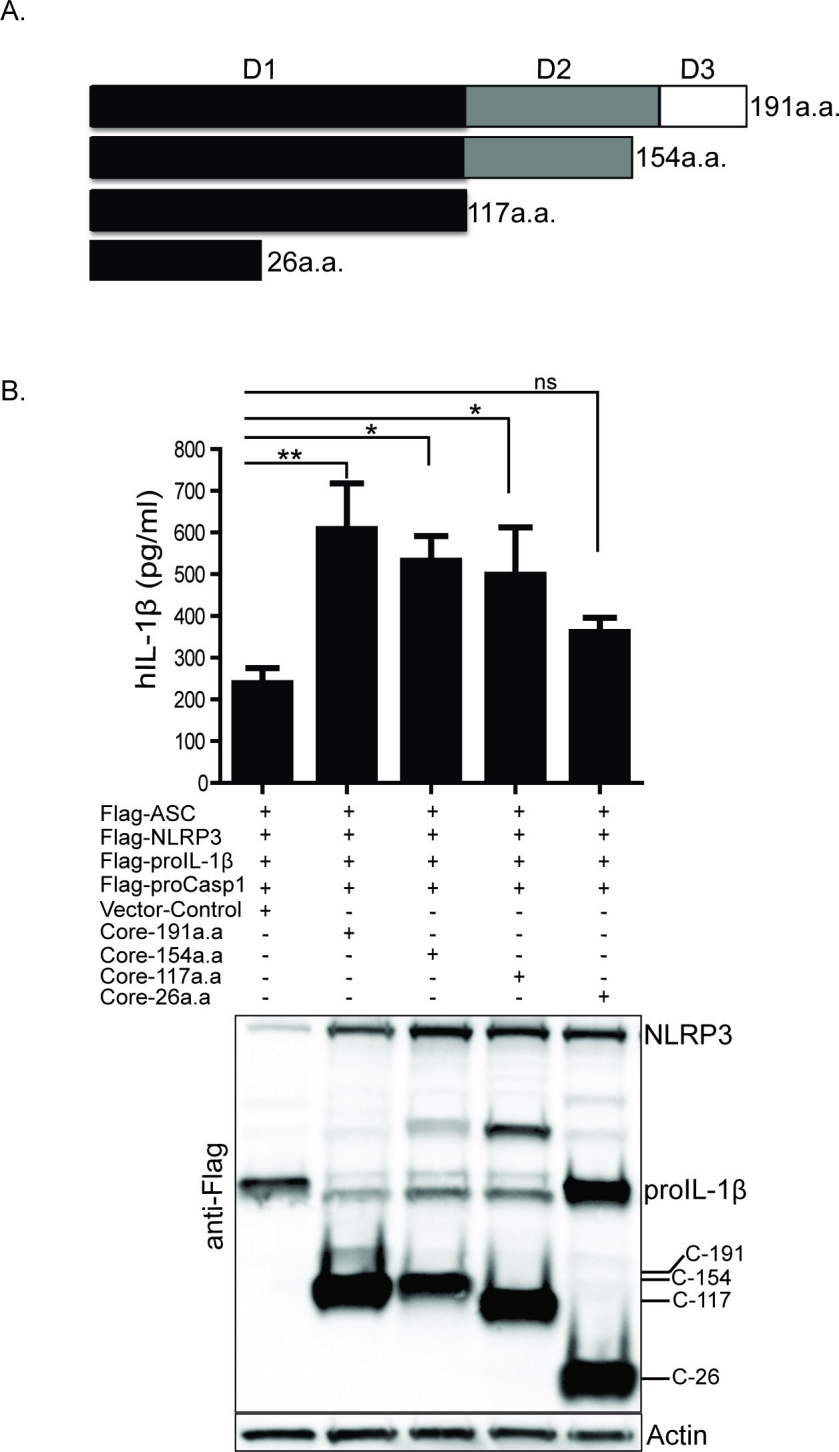
Full length of domain-I of HCV core protein is required for optimal NLRP3 inflammasome activation. (A) A schematic of HCV core protein major domains. (B) ELISA of IL-1β in the reconstituted cells transfected with various c-terminus truncation mutants of HCV core protein. Anti-Flag antibody stains the core constructs and actin serves as a loading control. Experiments were performed with three replicates and are representative of three independent experiments. Data are represented as means and +/- SD, *P<0.05, **P<0.01 and ns = non-significant.

The HCV core protein is expressed from the viral polyprotein as a 191-amino acid polypeptide [57, 58]. The full-length core protein is composed of major domain-I and domain-II. Domain-I comprises the basic N-terminus region that binds viral RNA and is involved in self-oligomerization [57, 59]. Domain-II comprises the hydrophobic region of the protein that mediates association with membrane and lipid droplets. The minor domain-III comprises the C-terminal region and the signal sequence for cellular localization of E1 wherein it is cleaved off by signal peptide peptidase during polyprotein processing [57]. To define the domain of HCV core that is required for NLRP3 inflammasome activation, we tested a panel of core truncation mutants for their ability to induce NLRP3 inflammasome activation in the reconstituted U2OS cell system (Fig 5A). HCV core constructs lacking Domain-II and domain-III retained the ability to stimulate IL-1β processing and release similar to full-length core protein, revealing that NLRP3 activation is directed by core protein domain-I (Fig 5B). Truncation of domain-I to a 26 aa N-terminal portion resulted in a loss of NLRP3 activation. Overexpression of the 26 aa N-terminal protein resulted in no processing of IL-1β similar to vector control (Fig 5B, lower panel), which is evident by the presence of more proIL-1β band in the western blot. Therefore, the full domain-I region is necessary and sufficient for induction of NLRP3 inflammasome activation by HCV core protein.

**Fig 6:**
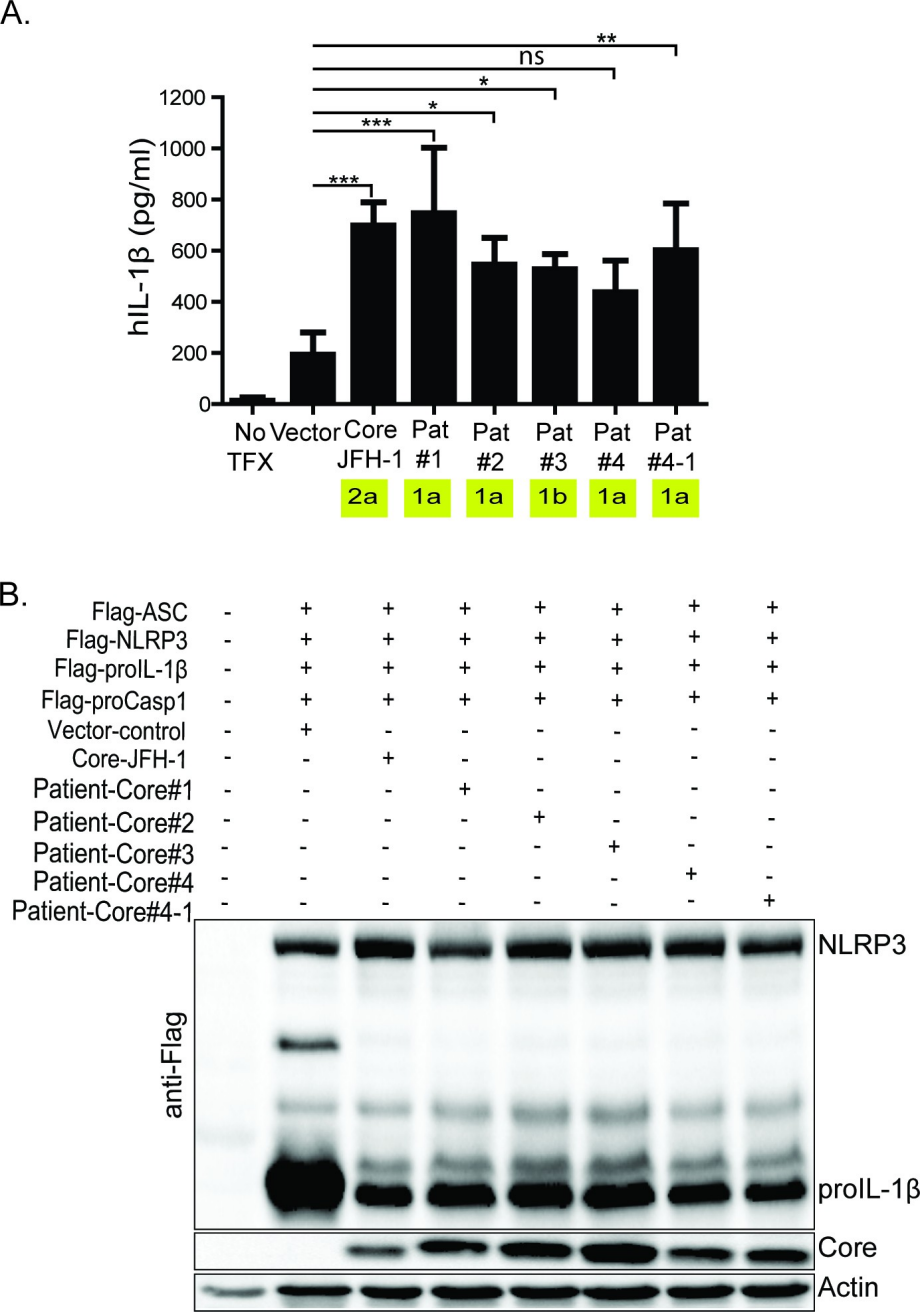
Core from HCV infected patients variably stimulate NLRP3 inflammasome activation. (A) IL-1β ELISA. HCV core, from acutely infected individuals, expressing constructs co-transfected with NLRP3 inflammasome components. (B) western blot depicting the expression of the patient-derived core as well as the inflammasome components. Experiments were performed with three replicates and are representative of four independent experiments. Data are represented as means and +/- SD, *P<0.05, **P<0.01, ***P<0.001 and ns = non-significant.

We next tested the ability of four patient-derived HCV core protein constructs to trigger NLRP3 inflammasome activation. Each of the clinical isolates of the core-coding region of HCV was recovered from sera of patients undergoing acute HCV infection with HCV genotype 1 prior to antiviral therapy, with each patient progressing to chronic infection [60]. For patient-4, sera samples were collected at two time points, at month-0 and month-2 post-HCV infection. HCV core protein from each patient stimulated NLRP3 inflammasome activation for IL-1β processing and release when expressed in reconstituted U2OS cells (Fig 6A and 6B). Interestingly, patient 4 core protein sequence displays variable activity to induce the NLRP3 inflammasome, and this core protein lacked the ability to significantly trigger IL-1β release over vector control while core sequence 4–1, isolated two months later, triggered significant IL-1β release. Examination of the sequence of each core protein construct from patient 4 revealed the acquisition of an amino acid substitution in domain-I within core 4–1 that links with inflammasome activation ([60] and S3 Fig). Thus, sequence diversity among core domain-I might impact NLRP3 activation potential of the HCV core protein.

**Fig 7:**
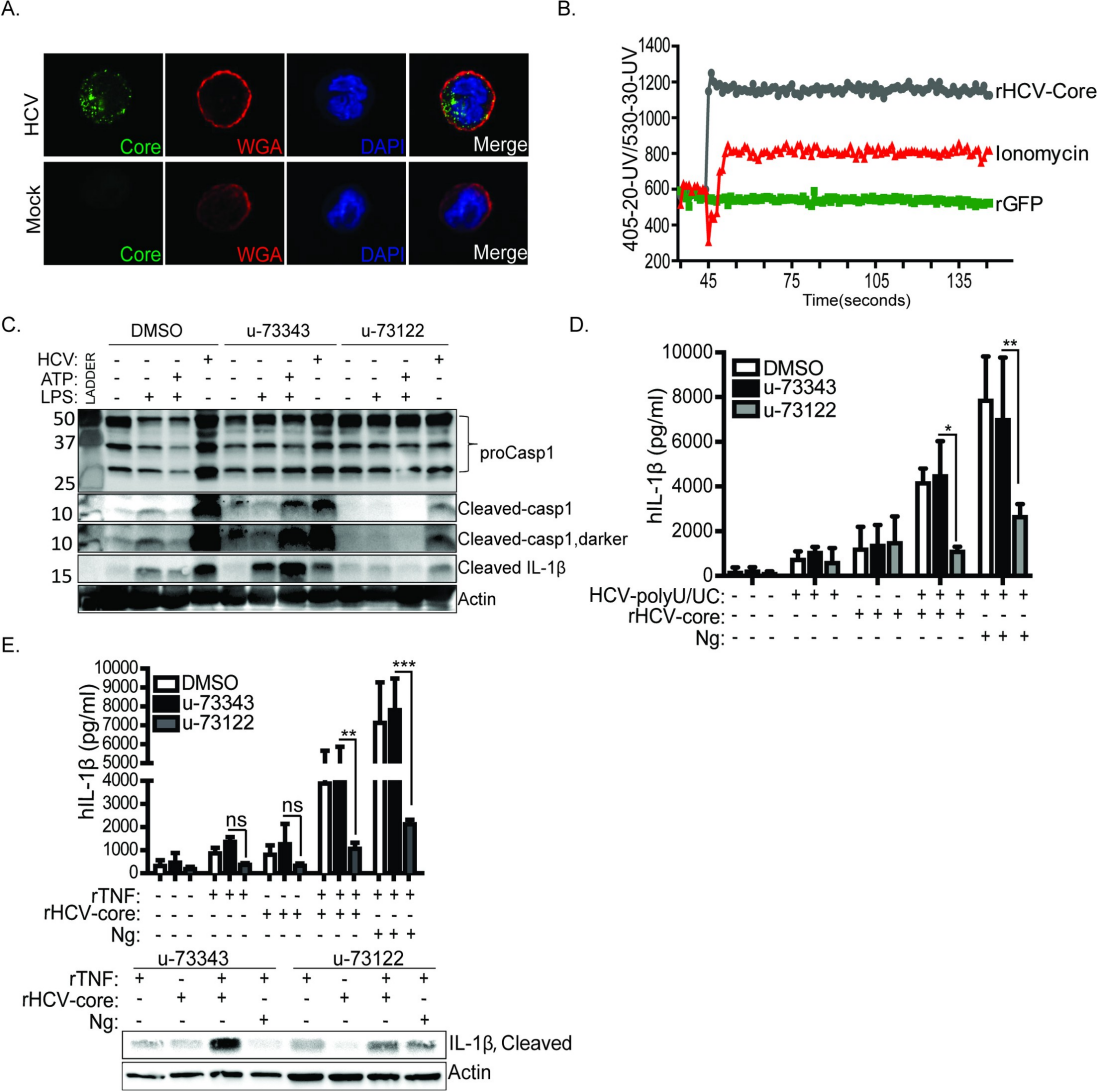
HCV core protein modulates cellular calcium to stimulate inflammasome activation and IL-1β release. (A) Wheat germ agglutinin staining of THP-1 cells treated with HCV. THP-1 cells were exposed to HCV 30-60minutes. Before fixing with paraformaldehyde, cells were labeled with wheat germ agglutinin to mark the plasma membrane. Red stains the plasma membrane (wheat germ agglutinin), green stains HCV core protein within THP-1 cytoplasm and blue (DAPI) stains the nuclei. (B) Calcium flux in THP-1 cells post stimulation with rHCV-Core or ionomycin or rGFP. (C) immunoblot showing the presence and absence of cleaved IL-1β and caspase-1 in HCV treated cells in the presence of u-73343 (inactive phospholipase C inhibitor) or u-73122 (active phospholipase C inhibitor, control) or DMSO (vehicle control). (D) ELISA of IL-1β in THP-1 cells post stimulation with HCV polyU/UC and rHCV-core or HCV polyU/UC and Ng in the presence of u-73122 or control u-73343. (E) TNF primed THP-1 cells were treated with rHCV-core or Ng in the presence and absence of calcium inhibitor (u-73122) or inactive analog (u-73433) or vehicle control (DMSO). Lower panel of (E) shows immunoblot of active IL-1β protein post treatment with calcium inhibitor. Experiments were performed in duplicates and are representative of three or more independent experiments. Data are represented as means and +/- SD, for (D) and (E) *P<0.05, **P<0.01, ***P<0.001 and ns = non-significant.

To determine the mechanism by which HCV core protein triggers IL-1β production, we first assessed the subcellular distribution of the core protein after virion uptake of HCV-treated THP-1 macrophages. We found that within one hour following exposure to HCV, the core protein was present within the cell cytoplasm. HCV core protein was also punctuated around the plasma membrane, likely representing cell surface-bound virion [27] (Fig 7A). We examined the possibility of core interacting with any of the NLRP3 inflammasome components as a mechanism of core-induced IL-1β production, but we observed no interaction between core and any of the NLRP3 inflammasome components (S4A Fig). Among other actions of the HCV core protein, cytoplasmic core protein modulates calcium flux and calcium-dependent signaling in infected hepatocytes [61]. Ion flux, including calcium and potassium, are major contributors of NLRP3 inflammasome activation [62–67]. We therefore measured calcium mobilization in macrophages treated with purified rHCV-core as a potential mechanism of NLRP3 inflammasome activation. Treatment with rHCV-core but not rGFP (control) induced a rapid increase of intracellular calcium both in primary monocytes-derived macrophages and THP-1 macrophages (Figs 7B and S4B). As phospholipase C is a major regulator of intracellular calcium mobilization, we then treated THP-1 macrophages with DMSO (vehicle control), the phospholipase C inhibitor (u-73122) [68] or an inactive inhibitor analog (u-73343) in the presence of HCV or rHCV-core. HCV triggered caspase-1 activation and IL-1β processing in cells treated with DMSO or u-73343, but treatment with the active phospholipase inhibitor u-73122 prevented caspase-1 activation and IL-1β processing (Fig 7C). Importantly, we found IL-1β production induced by rHCV-core treatment of THP-1 macrophages is specifically suppressed by treatment of cells with u-73122 phospholipase C inhibitor (Figs 7D, 7E and S4C) as treatment with the control Ng. As shown above (see S2 Fig), treatment with TNF-α stimulates signal-one activation, and we tested if inhibition of phospholipase C by u-71322 treatment did not block signal-one priming of the NLRP3 inflammasome. When we reversed the treatment sequence by stimulating with TNF-α first then treating with the phospholipase C inhibitor, IL-1β production was similarly suppressed (S4D Fig). These results collectively indicate that the HCV core protein imparts an activating signal for IL-1β production and release from macrophages through induction of phospholipase C-mediated calcium mobilization that activates the NLRP3 inflammasome.

## Discussion

**Fig 8:**
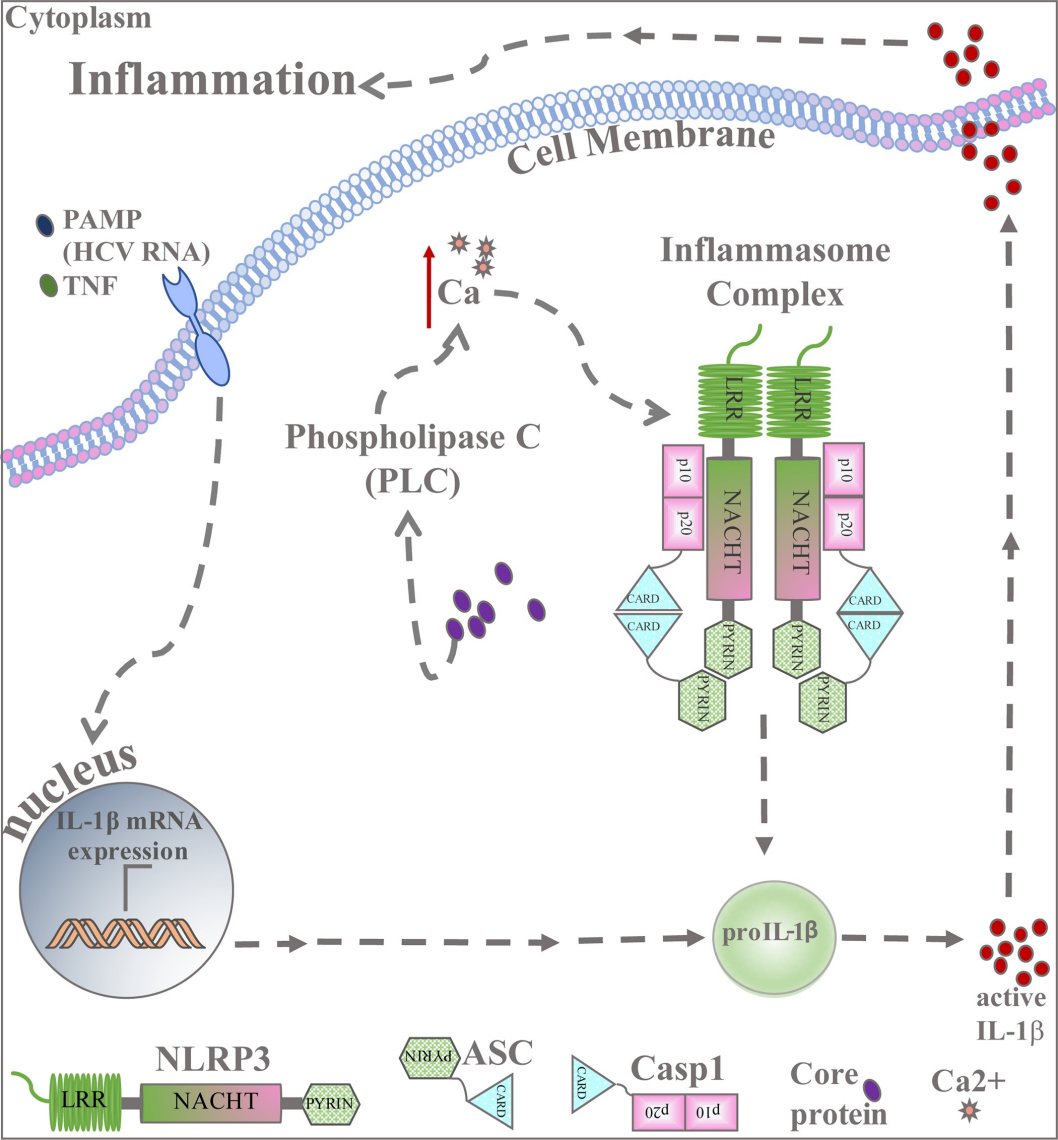
HCV Core-mediated IL-1β production in macrophages. A working model of how HCV-mediated inflammation is propagated by HCV core protein in macrophages. Macrophages priming by TNF and/or viral PAMPs such as viral RNA leads to signal-one induction via NFκB mediated signaling. Macrophage engulfment of HCV leads to core accumulation in the cytoplasm, which stimulates the activation of the NLRP3 inflammasome. Core triggered NLRP3 activation is induced by HCV-core-mediated calcium mobilization through phospholipase C-dependent pathways.

Our findings defines the molecular mechanism by which HCV triggers IL-1β production in macrophages (Fig 8). Hepatic macrophages effectively phagocytose macromolecules in the liver to continually survey the hepatic environment and respond to microbial threats [16]. Within the HCV-infected liver, virus-derived antigens, viral RNA and/or viral protein and other inflammatory mediators such as TNF-α can serve as priming factors in hepatic macrophages leading to NFκB activation and proIL-1β production [27, 69]. Our observations demonstrate that the resulting signal-one primed macrophages are then responsive to HCV core protein both within the virion and as a cell-free protein present in patient blood [70]. HCV virion or core protein uptake then deposits the viral core protein in the cell cytoplasm where it induces phospholipase C-mediated calcium flux leading to NLRP3 inflammasome activation, thus establishing the hepatic inflammatory environment.

Calcium signaling is a complex event that is important for many cellular processes including activation of the NLRP3 inflammasome [62, 65, 67, 71]. It has been shown that the dynamics of potassium efflux and calcium influx, and subsequent increase in intracellular calcium, leads to mitochondria damage, which releases reactive oxygen species that ultimately activate NLRP3 inflammasome [65]. Our finding that HCV, through the action of its virion core protein within macrophages, modulates intracellular calcium flux/mobilization through a phospholipase C-dependent process, suggests that the HCV core protein may modulate the expression of calcium-sensing receptor (CaSR) or other components within a phospholipase C-regulated pathway [72, 73]. CaSR is expressed in macrophages, including THP-1 cells and its expression is enhanced upon calcium binding wherein it may promote further accumulation of intracellular calcium following stimulation by the core protein [71–73]. Alternatively, the subcellular localization within cytoplasmic compartments of the macrophage might dictate the activity of HCV core to impart inflammasome activation. For example, the HCV core protein has been shown to associate with cytosolic membrane compartments including the endoplasmic reticulum membrane in contact with lipid droplets. Core insertion within these membranes might also lead to calcium "leakage" or depletion to evoke CRAC channel activity and Ca influx [72]. While further studies need to delineate the role of other pathways that mediate intracellular calcium increase in core-induced IL-1β production, our observations indicate that phospholipase C-mediated calcium production/mobilization directed by the viral core protein is an essential process of HCV-induced NLPR3 inflammasome activation following virion or core protein uptake by macrophages.

Our studies show that KCs, THP-1 macrophages, and primary macrophages produce and secrete inflammatory IL-1β following exposure to HCV [27] or soluble viral core protein (this study). Although macrophages do not support HCV replication, the sensing of viral-derived antigens could occur at multiple places initiated by the process of virion uptake by macrophages such as phagocytosis-mediated engulfment. Our studies show that the HCV virion itself comprises the mediators to drive both signal-one and signal-two of NLRP3 inflammasome activation. Just as inflammatory cytokines, such as TNF-α within the blood and hepatic environment, can mediate signal-one triggering in responsive macrophages, engagement of TLR7 by the viral RNA within the internalized HCV virion serves as a potent signal-one inducer [27]. Moreover, our current findings indicate that poly-U/UC RNA of the major PAMP of HCV recognized by RIG-I [52, 74] can trigger signal-one to prime macrophages. Thus, multiple stimuli can prime the macrophage for a response to HCV core protein in order to trigger NLRP3 inflammasome activation. As a result, hepatic macrophages produce and release IL-1β and propagate the hepatic inflammatory response that underlies liver inflammation and disease in chronic HCV infection.

p7 could also play an important role in hepatic inflammation. As previously reported [19, 50], and consistent with our data sets (see Fig 2A) p7 can impart NLRP3 inflammasome activation. p7 is a viroporin that has ion channel activity [75], wherein p7-mediated ion-flux might impart NLRP3 inflammasome signal-two. However, our results argue against p7 activity having a major role in inflammasome activation induced by the incoming HCV virion, as treatment with a p7 ionophore inhibitor had no effect upon virion-induced NLRP3 activation. Instead, we propose that within an infected liver, macrophage engulfment of infected hepatocytes undergoing active HCV replication might be more likely to result in p7-directed inflammasome activation.

HCV employs many innate immune evasion strategies to mediate persistent infection linked with chronic liver disease [76]. In infected hepatocytes, HCV blocks viral poly-U/UC PAMP/RIG-I-mediated production of the type 1 and 3 interferon (IFN) through the action of the viral NS3/4A protease, which targets and cleaves MAVS to abrogate intracellular antiviral defenses [77]. Furthermore, the HCV core protein is known to antagonize IFN signaling pathways preventing the expression of antiviral effector genes [78]. Moreover, IFN negatively regulates the NLRP3 inflammasome through anti-inflammatory IL-10 [79]. These processes collectively dampen the establishment of an effective antiviral state and provide a suitable platform for persistent infection, IL-1β production, and hepatic inflammation that contribute to ongoing immune-mediated liver injury and hepatitis.

Our study underscores a central role of macrophages in HCV pathogenesis. IL-1β is a major mediator of hepatic inflammatory cytokine production from liver cells responding directly to IL-1β signaling [80]. Blockade of IL-1β production with anti-IL-1β agents may offer an attractive therapeutic option for HCV infected individuals. While treatment with DAAs present a cure for HCV infection and can restore hepatic innate immune and inflammatory homeostasis in the liver [81, 82], studies show that hepatic inflammation and altered inflammatory cytokine levels still persists in some patients successfully treated with DAAs who display ongoing liver injury [12, 83–85]. These studies highlight sustained inflammatory cytokines such as IL-1β and IL-1β responsive cytokines as components linked to residual sustained liver disease from hepatic injury after HCV infection. The IL-1β /NLRP3 inflammasome cascade [86] may therefore present an attractive target for treating liver disease induced by HCV and other causes of liver inflammation.

## Materials and method

### Reagents

Antibodies: monoclonal anti-HCV core antibody (ThermoFisher), anti-influenza-M2 and anti-Caspase-1 (Santa Cruz Technologies), anti-Flag (Sigma), anti-actin (Millipore), anti-IL-1β (Cell Signaling, detects only cleaved IL-1β protein), goat-anti-mouse or goat-anti-rabbit secondary antibodies (ThermoFisher). HCV E2 antibody (Austral Biologicals). Other reagents: Recombinant human TNF and m-CSF were purchased from Peprotech and recombinant HCV core protein (rHCV-core) (Meridian Life Science, Inc). The recombinant core protein is produced in *Pichia pastoris* and was used at 10-30ug/ml. Nigericin, ATP, D609, u-73343, u-73122, DMSO, and PMA (Sigma). Indo-I-AM calcium flux detection kit was purchased from Calbiochem and LPS from Addipogen. For polyU/UC RNA transfection and/or DNA transfection, Mirus Trans-IT mRNA transfection (for RNA transfection) and Mirus low-toxicity (LT1) (for DNA transfection) were used. The polyU/UC RNA was made as previously described [52]. The p7 inhibitor (JK3/32), which is the active analog, and (R-21), which is the inactive analog, were dissolved in DMSO and used at 1-10uM with final DMSO content of 0.25%. For all kits used, the manufacturer’s protocol was followed and if a modification is made, it is noted.

### Tissue culture

THP-1 cells were obtained from ATCC, U2OS (osteosarcoma cells) were obtained from ATCC, and Huh7 K2040 cells and Huh7.5 cells were described previously [87]). NLRP3 knockout cells or non-targeting controls in THP-1 cells were made using CRISPR (Kindly generated by Andrey Shuvarikov, University of Washington). Cells were maintained in RPMI-1640 for THP-1 and DMEM for U2OS cells. All media were supplemented with 10% FBS, 0.01M Hepes, 1mM sodium pyruvate, 2mM L-glutamine, antibiotics and non-essential amino acid at 1x. All cells were maintained at 37°C under 5% CO_2_. For differentiating THP-1 cells into macrophages, THP-1 monocytes were treated with 40nM of PMA for 24hrs. The following day the cells were washed with PBS and then cultured in fresh media for another 24hrs for resting. Stimulation of THP-1 cells took place after resting (48hrs after initial PMA treatment).

### HCV preparation and propagation

HCV was propagated and tittered as described previously [88]. Briefly, Huh7.5 [49] were inoculated with HCV. Virus was removed after one hour of inoculation and then replaced with fresh media. 48hrs post infection, culture media containing virus was removed and replaced with fresh media. Infected cells were then cultured for 3–5 days. At the day of harvest, the infectious supernatant was filtered through 0.1um to remove cell debris and concentrated the virus using the Millipore virus concentrating centricons. After concentrating, the virus stock was aliquoted and stored at -80°C. The virus titration analysis was performed on Huh7.5 using the NS5A monoclonal antibody (kindly provided by Dr. Charles Rice, The Rockefeller University). The HCV pseudoparticles (HCVpp) and VSVpp (kindly provided by Dr. Jane McKeating, University of Oxford). For ultraviolet inactivation of HCV, the virus was crosslinked using the uv stratalinker (1200mJ/cm^2^ over 1800 seconds). For measuring JFH-1 inhibition by JK3/32, Huh7 cells were cultured and propagated as described previously [51]. Secreted infectivity was measured as described [89]. Briefly, 1ug of linearized HCV construct pJFH-1 was used as a template for *in vitro* transcription (RiboMax express, promega) as per manufacturer’s instructions. RNA was then purified by acid phono/chloroform and isopropanol precipitation. 4x10^6^ Huh7 cells were then electroporated as described previously [51]. Electroporated cells were seeded at 2.5x10^4^ cells/well in 100ul volume in 96 well plates and incubated for 4hrs prior to addition of inhibitor. Compounds were prepared at 400x in DMSO, diluted 1:20 into media in an intermediate plate, and 1:20 into the final cell plate to yield final 0.25% (v/v) DMSO. All compound treatments were dosed in duplicate. Treated cells were incubated 72hrs before performing 1:4 dilution (50ul) of virus-containing supernatant onto a plate of naïve Huh7 cells (150uls), seeded at 8x10^3^cells/well 6hrs previously. Infected Huh7 cells were incubated 48hrs before washing 3x in PBS and fixing in 4% PFA. Fixed cells were washed in PBS and permeabilized using 0.1% triton X-100 (v/v) in PBS for 10min, room temperature. Following PBS wash, cells were immune-stained for NS5A to quantify infected cells. Sheep anti-NS5A antibody [90] was used at 1:2000 in PBS supplemented with 10% FBS, 16hrs at 4°C. Following 3x PBS washes, Alexafluor594 nm Donkey anti-Sheep antibody (Invitrogen) was added at 1:500, 2hrs in the dark. Cells were washed twice in PBS and imaged using phase and red channels (IncuCyte ZOOM). Infected cells positive for NS5A expression were quantified using IncuCyte parameters previously described [89], normalized to DMSO controls.

### Plasmids and transfections

For the inflammasome components, the cDNA of human proIL-1β, procaspase-1, NLRP3 and ASC were purchased from Origene. These cDNAs were subcloned into pEF vector which contains the human elongation factor-1 alpha (EF1α) promotor to drive ectopic gene expression. The inflammasome components are Flag-tagged for easy detection. Viral proteins were subcloned and expression was detected either by Flag antibody or gene-specific antibody. To amplify the core region from the pSJ [88] (HCV JFH-1 sequence, genotype-2a) plasmid, the following primers were ordered from IDT: Core-forward primer: 5’gcgcgcggccgcatgagcacaaatcct3’, Core-reverse-primer: 5’gcgcggatccagcagagaccggaacggt3’. For p7 (genotype-2a) and M2, a minigene was ordered and cloned into either the pEF vector or the polycistronic pRRL-MND-vector (kindly provided by Dr. Daniel Stetson, University of Washington). For influenza M2 ion channel protein, the sequence of influenza A virus (A/Wyoming/03/2003(H3N2), accession number: DQ849011.1) was obtained from NCBI. The JFH-1-E1E2 polyprotein expressing plasmid (generously provided by Dr. Jane McKeating, University of Oxford). The patient core expressing constructs (kindly provided by Dr. Stephen Polyak, University of Washington). To achieve inducible inflammasome response in the U2OS-reconstituted system the following amounts of plasmids were co-transfected: 1ng of ASC, 1ng of procaspase-1, 100ng of NLRP3 and 100ng of proIL-1β. All plasmids including the viral protein expressing constructs or vector control were transfected at the same time. Samples were collected at either 24 or 48hrs post-transfection for ELISA and immunoblot analyses. For overexpression of HCV core or Flu-M2 in THP-1 cells, Lentiviral transduction method was employed. To generate Lentiviruses expressing HCV core or Flu-M2 or empty vector, the pRRL-MND-2A-eGFP-2A-Puro Lentiviral vector was used. The Lentiviruses were generated in 293 by transfecting the Lentiviral vector expressing gene of interest, pVSV expressing envelope and packaging vector pSPAX2. 24hrs post transfection, fresh media was added and then collected lentiviral particle containing supernatant at 48hr post media change. The Lentiviral particles were filtered to remove cell debris before proceeding with transduction. To generate stable cells expressing the desired transgene, 5-10x10^6 THP-1 cells were transduced in 10cm dish and then selected with puromycin. Plasmids and associated materials can be provided upon request.

### Western blot analysis and immunoprecipitation

For western blot, cell pellets were collected and washed once with 1x PBS. Then cells were lysed in modified RIPA buffer (50mM Tris-HCl, pH-7.5, 150mM NaCl, 0.1 SDS, 1% triton-x/NP-40, 1% Na-deoxycholate, 5mM EDTA) supplemented with protease inhibitor cocktail. Protein from cell lysates were resolved by SDS-PAGE with either commercially purchased precast gels (Biorad) or in lab made-15% or 12.5% gels and then transferred onto PVDF membrane. Probing for the indicated protein was performed using a specific antibody or anti-Flag and actin was used as a loading control. For immunoprecipitation (IP), cells were lysed in RIPA buffer. Cell lysate were incubated with anti-Core antibody or IgG control overnight at 4°C. The following day, IP was performed using protein G dynabeads and then immunoblot was performed as described.

### Quantitative real time polymerase chain reaction

RNA from cells was extracted using the RNeasy mini kit (Qiagen). After obtaining RNA, cDNA was made using the Biorad Iscrip Select cDNA synthesis kit. Then real time-PCR (qRT-PCR) was performed using SYBR green PCR master mix. IL-1β and GAPDH (housekeeping gene) primers were used (both purchased from Qiagen).

### Microscopy and confocal imaging

Cells were seeded on cover slips. After stimulation is complete, cells were washed with 1xPBS and then fixed with either 3% paraformaldehyde or ice-cold methanol. After fixation, cells were permeabilized with 0.2% of triton-x. Non-specific binding was blocked with 3% non-fat incarnation milk in 1x PBS. Staining with primary antibody was performed for 30min at room temperature. Alexa-Fluor-594 or alexa-Fluor-488 were used as secondary antibodies. The nucleus is stained with DAPI and wheat-germ agglutinin-594 (ThermoFisher) marks the plasma membrane. Imaging was performed using Nikon Eclipse Ti confocal microscope. Images were taken with 60X oil immersion objective. All images were mounted using the prolong gold antifade reagent. Images were acquired and processed using the NIS advanced research software.

### Measuring intracellular Calcium flux using Indo-I-AM

Calcium flux was measured in differentiated THP-1 cells. 2-5x10^6 cells were first stained with Indo-I-AM at 1:500 in 200ul-RPMI for 45min at 37°C. after staining with Indo-I, cells were washed with RPMI and either left untreated or stimulated with recombinant core for 10min-1hr. Samples were run on LSRII under the following settings: SS-355, FS-231, DAPI-A-352 and Hoechst-red-A-368. For baseline calcium levels, untreated cells were acquired for 10–30 seconds then stimulated cells were acquired for 2-3min. Calcium flux is represented as a ratio of DAPI to Hoechst-red. Calcium flux represents a shift from 530/30-uv-Hoechst-red (Blue, Ca-unbound) to 405/20-uv-DAPI (Violet, Ca-bound).

### Preparation of primary monocytes-derived macrophages

Peripheral blood mononuclear cells (PBMCs) of healthy donors were obtained from TRIMA leukoreduction (LRS) chambers (Bloodworks Northwest, WA). Total mononuclear cells were obtained by centrifugation using ficoll-paque plus (followed manufacturer’s protocol). Briefly, the whole blood was diluted 1:2 with 1xPBS then 35ml of the diluted blood was layered on top of 15ml of Ficoll-paque plus in 50ml conical tube. Centrifugation was performed at room temperature at 300xg for 30-45min with no brake. After centrifugation is complete, 5–10 million total PBMCs were cultured in complete-RPMI containing 40-100ng/ml of human m-CSF. Two days after culturing, non-adherent cells were removed and fresh RPMI containing 40-100ng/ml of m-CSF was added. After five days of culturing, cells were collected by scrapping gently in RPMI then seeded 0.3–0.8 million cells per well of a 12-well plate. The following day cells were treated with different stimuli.

### Ethics statement

The human blood samples were obtained from healthy donors. The samples were purchased from Bloodworks Northwest (Washington). The samples are delivered in TRIMA leukoreduction (LRS) chambers and then processed to isolate PBMCs. Samples were non-identifiable.

### ELISA

For ELISA, supernatant from both non-stimulated and stimulated cells were collected. To remove cell debris, cells were spun for 5 minutes at medium speed. ELISA was performed according to the manufacturer’s manual (Biolegend, Inc.).

### Statistical analysis

For statistical analysis, student t-test or one-way Anova (for multiple comparisons) was performed. Any p value less than 0.05 was considered statistically significant. Graphs are represented as the means +/- SD.

## Supporting information

S1 Fig (A) IL-1β ELISA in THP-1 cells. THP-1 cells were differentiated with PMA then treated with uv-HCV or LPS/Ng in the presence of a pharmacological p7 ion channel inhibitor. Jk3/32 (active inhibitor), R-21 (inactive inhibitor) and DMSO (vehicle control). (B) JHF-1 secretion post incubation in either JK3/32 or DMSO control.
(TIF)

S2 Fig qRT-PCR of IL-1β in PMA differentiated THP-1 cells post stimulation with TNF-α at 1ug/ml. Induction of IL-1β gene expression is normalized to GAPDH.
(TIF)

S3 Fig Amino acid core protein sequence alignment derived from HCV infected individuals, isolated and cloned from HCV infected patient sera at the acute phase [60]. Patient#1(ID: 4-7-1), patient#2 (ID: 27-9-5), patient#3(ID: 3-9-5), patient#4(ID: 6-8-2) and patient#4-1(ID: 30-4-4).
(TIF)

S4 Fig (A) Immunoblot showing Co-IP of core with NLRP3 inflammasome components in the reconstituted system. (B) Intracellular calcium in primary human monocyte-derived macrophages upon treatment with rHCV-Core. (C) ELISA of IL-1β in differentiated THP-1 cells stimulated with rTNF and rHCV-core in the presence of D609 inhibitor. For (D) cells were first stimulated with TNF then treated with rHCV-core or ATP in the presence of DMSO or u-73343 or u-73122.
(TIF)
