# Supplementary figures and images for: Modulation of calcium signaling pathway by hepatitis C virus core protein stimulates NLRP3 inflammasome activation
Source: PLoS Pathog. 2019 Feb 27;15(2):e1007593. doi: 10.1371/journal.ppat.1007593 (PMC6392285; doi:10.1371/journal.ppat.1007593)

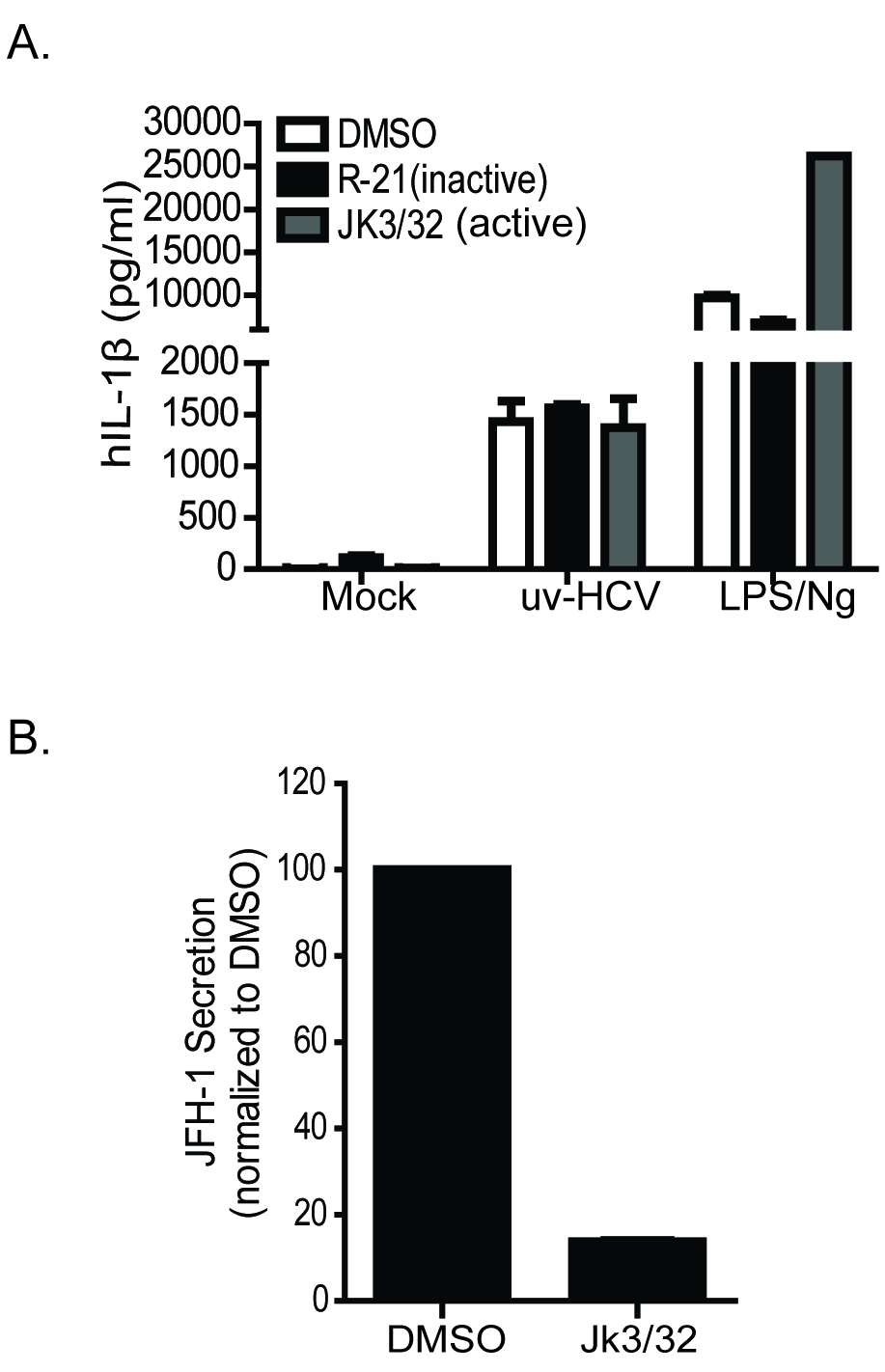

Supplement: S1 Fig — (A) IL-1β ELISA in THP-1 cells. THP-1 cells were differentiated with PMA then treated with uv-HCV or LPS/Ng in the presence of a pharmacological p7 ion channel inhibitor. Jk3/32 (active inhibitor), R-21 (inactive inhibitor) and DMSO (vehicle control). (B) JHF-1 secretion post incubation in either JK3/32 or DMSO control. (TIF) [file ppat.1007593.s001.tif]

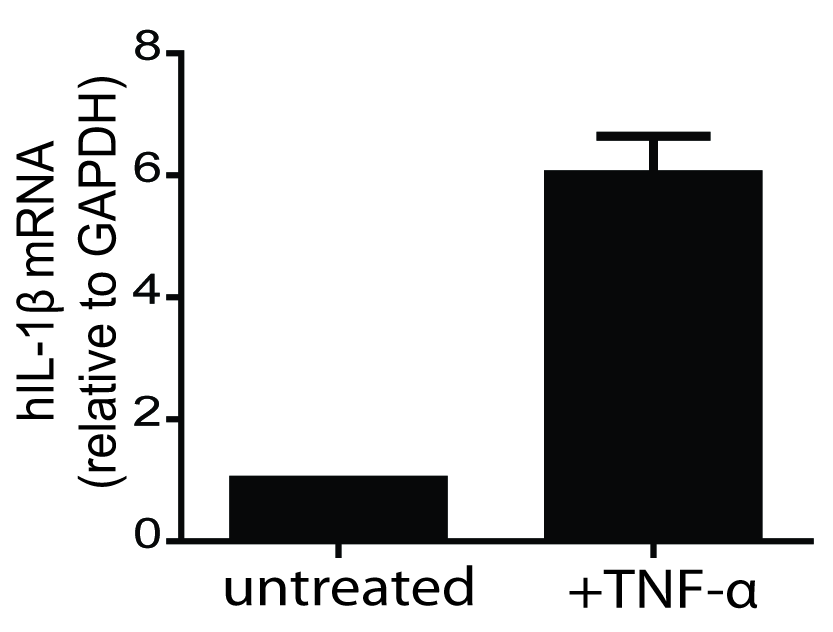

Supplement: S2 Fig — Induction of IL-1β gene expression is normalized to GAPDH. (TIF) [file ppat.1007593.s002.tif]

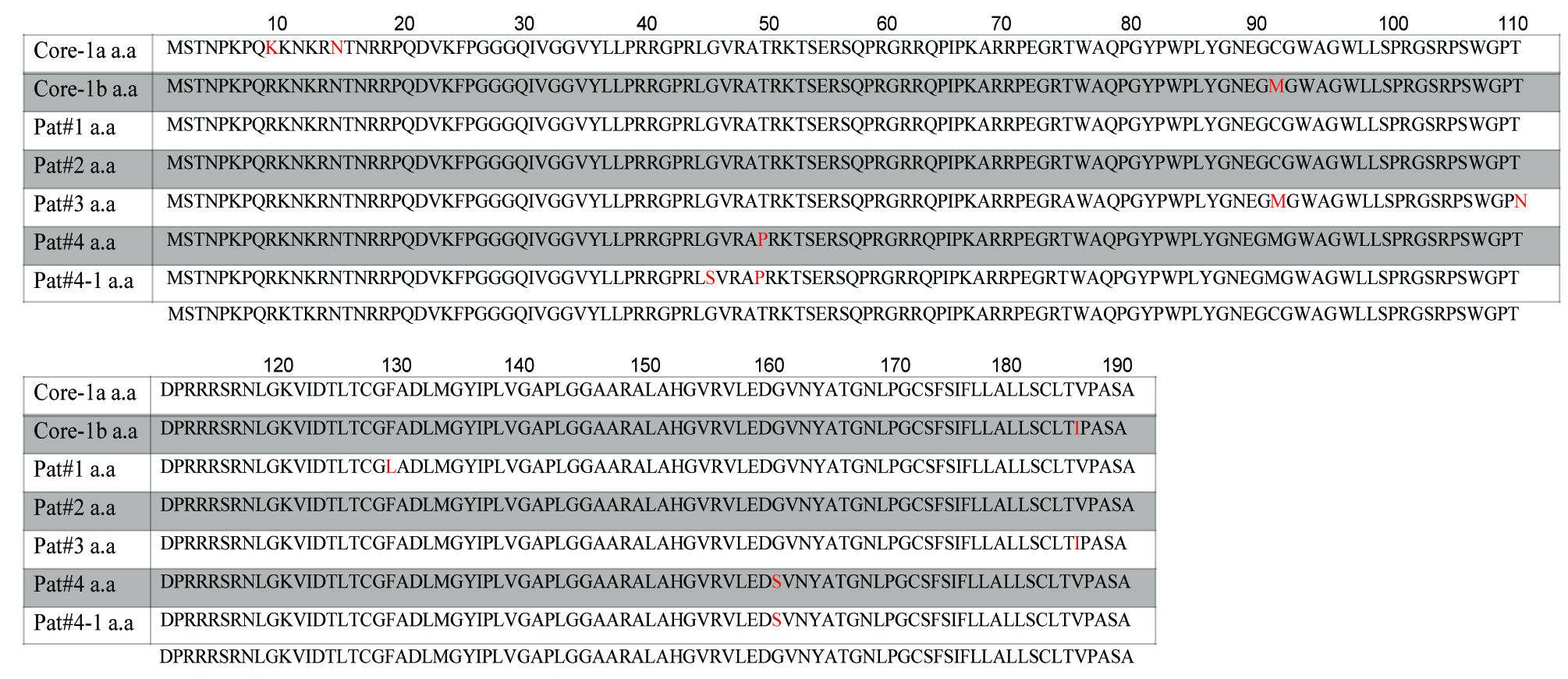

Supplement: S3 Fig — Patient#1(ID: 4-7-1), patient#2 (ID: 27-9-5), patient#3(ID: 3-9-5), patient#4(ID: 6-8-2) and patient#4-1(ID: 30-4-4). (TIF) [file ppat.1007593.s003.tif]

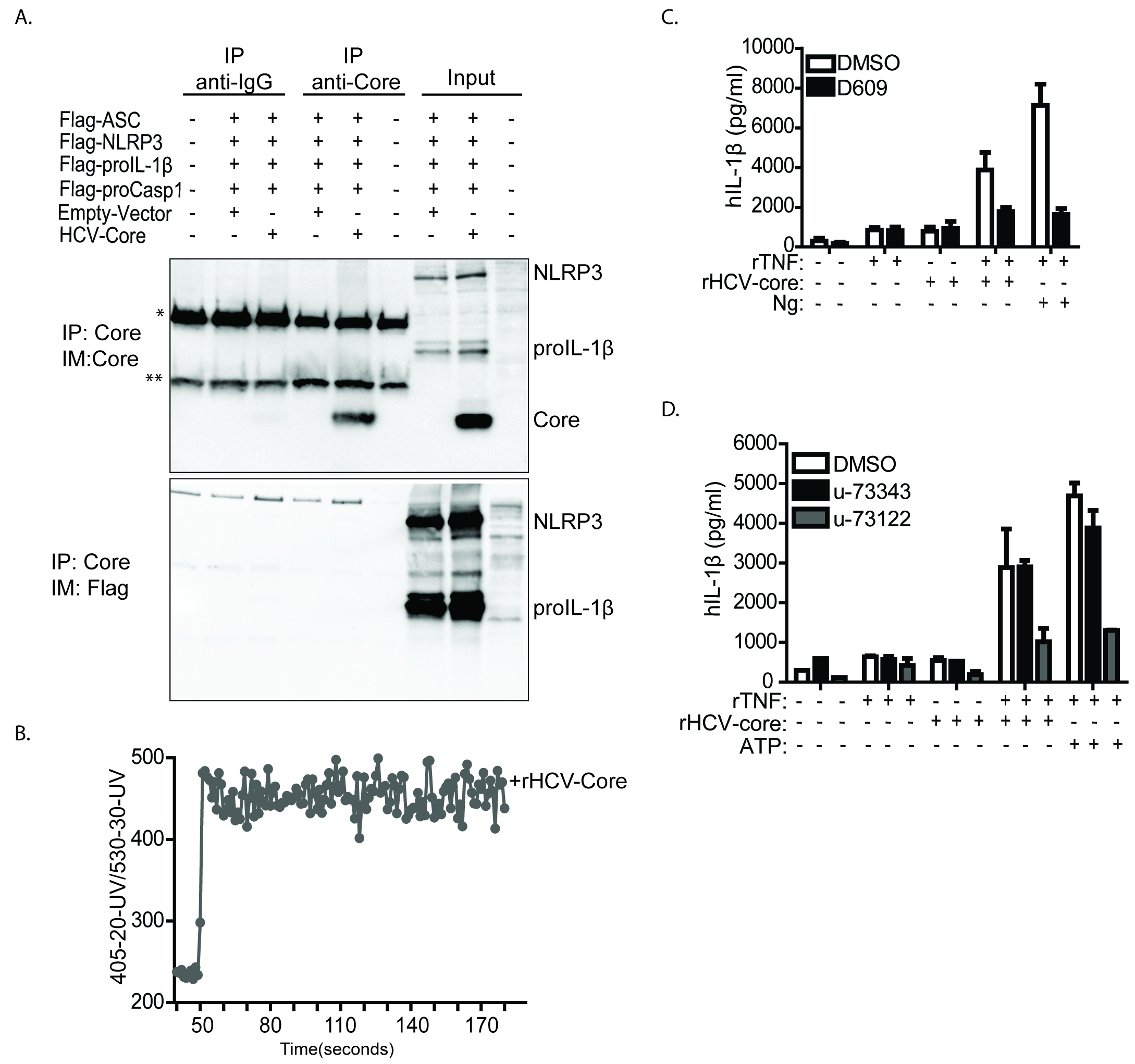

Supplement: S4 Fig — (A) Immunoblot showing Co-IP of core with NLRP3 inflammasome components in the reconstituted system. (B) Intracellular calcium in primary human monocyte-derived macrophages upon treatment with rHCV-Core. (C) ELISA of IL-1β in differentiated THP-1 cells stimulated with rTNF and rHCV-core in the presence of D609 inhibitor. For (D) cells were first stimulated with TNF then treated with rHCV-core or ATP in the presence of DMSO or u-73343 or u-73122. (TIF) [file ppat.1007593.s004.tif]
